# Primary synovial sarcoma and acinar adenocarcinoma of prostate rarely occur simultaneously: A case report

**DOI:** 10.1097/MD.0000000000036151

**Published:** 2023-11-24

**Authors:** Qichong Shi, Yun Zhou, Longmei Wang

**Affiliations:** a Department of General Surgery, Huai’an Fifth People’s Hospital, Huai’an, Jiangsu, China; b Department of Pathology, Hen’an University, Kaifeng, He’nan, China; c Department of Pathology, Huai’an Fifth People’s Hospital, Huai’an, Jiangsu, China.

**Keywords:** acinar adenocarcinoma, prostate, synovial sarcoma

## Abstract

**Rationale:**

Primary synovial sarcoma of the prostate is an extremely rare mesenchymal malignant soft tissue tumor with unique morphological features. Synovial sarcoma often occurs in the pararticular tissues of limbs in young people, but rarely occurs in prostate. Because it is very rare, it is easily misdiagnosed as benign prostatic hyperplasia or prostate cancer clinically. A case of synchronous acinar adenocarcinoma of the prostate has not been reported. In this article, we report a unique case of primary prostatic synovial sarcoma with acinar adenocarcinoma.

**Patient concerns:**

A 58-year-old male patient was found to have a prostate mass during physical examination. Prostate ultrasound examination showed an increase in prostate volume of 5.2 × 3.3 × 3.3 cm, mixed echo mass can be seen on the left side of the prostate, with a size of approximately 4.9 × 4.3 cm, left seminal vesicle compressed.

**Diagnoses:**

Prostatic synovial sarcoma (biphasic type) combined with prostatic acinar adenocarcinoma (Gleason 3 + 3).

**Intervention:**

The patient received radical prostatectomy, followed by adjuvant chemotherapy and radiotherapy.

**Outcome:**

After 2 months of follow-up, at the time of writing this article, the patient received a comprehensive treatment plan of adjuvant chemotherapy and radiotherapy for 2 months, and no recurrence or metastasis was found.

**Lessons:**

Primary prostatic synovial sarcoma (biphasic type) combined with prostatic acinar adenocarcinoma is a very unique and rare case, and effective treatment guidelines are not yet clear, posing new challenges to clinical treatment. Making full use of pathological and imaging examinations, early diagnosis and radical surgery combined with multidisciplinary treatment seem to be still a positive method.

## 1. Introduction

Synovial sarcoma is a malignant invasive tumor originating from soft tissue, accounting for 5% to 10% of all soft tissue tumors.^[1]^ It mainly occurs near the joints of limbs, and can occur in the pleura, lung, kidney, gastrointestinal tract, peritoneum, esophagus, and other parts.^[2]^ It rarely originates from the prostate gland. Because of its unique morphological characteristics, prostatic synovial sarcoma is usually found in the late stage and is easily misdiagnosed. Synovial sarcoma of the prostate has also been reported in the literature, but it is very rare to have prostate adenocarcinoma at the same time. We report a case of primary prostatic synovial sarcoma (biphasic type) with acinar adenocarcinoma in a middle-aged male.

## 2. Case presentation

**Figure 1.**
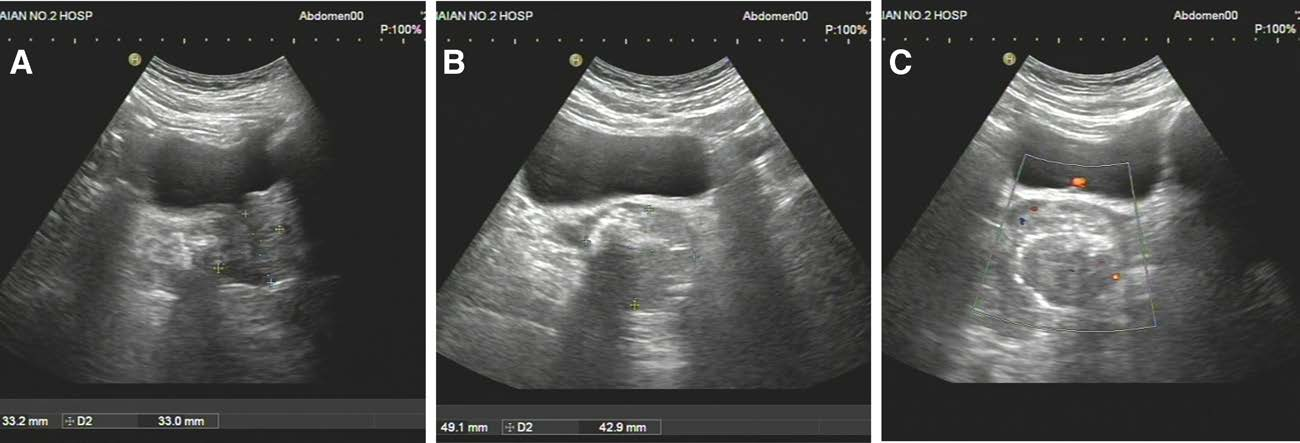
(A–C) Prostate ultrasound (US) showed an increase in prostate volume, with a mixed echogenic mass of approximately 4.9 × 4.3 cm in size visible on the left side of the prostate, most of which protrude outward from the contour, with irregular strong echoes visible inside, and local blood flow signals. The left seminal vesicle gland is compressed.

**Figure 2.**
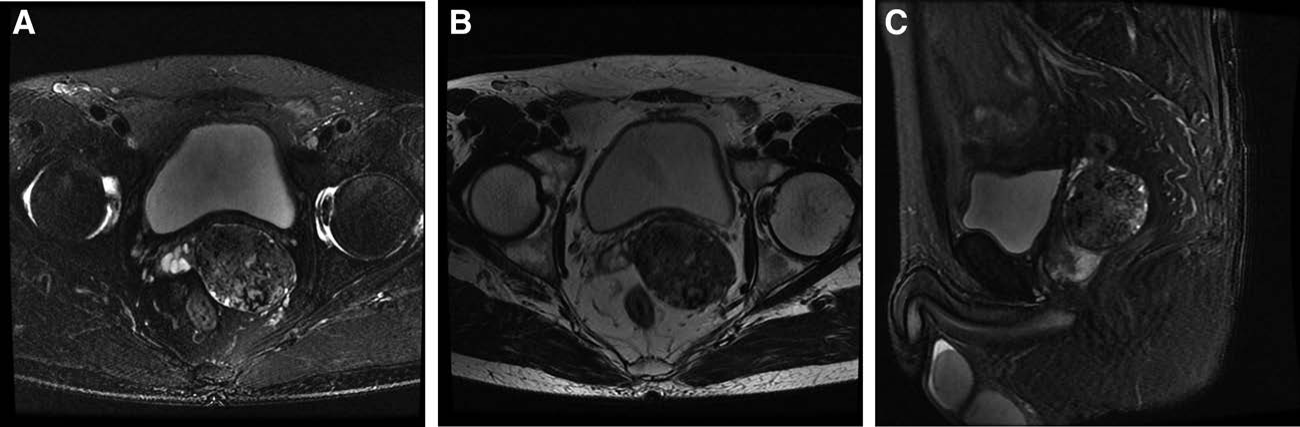
(A–C) Prostate magnetic resonance imaging (MRI) shows an increase in prostate volume, with T1 and T2 mass signals protruding to the left and rear in the left central zone of the prostate, and spotted high signal in DWI. DWI = diffusion weighted imaging.

**Figure 3.**
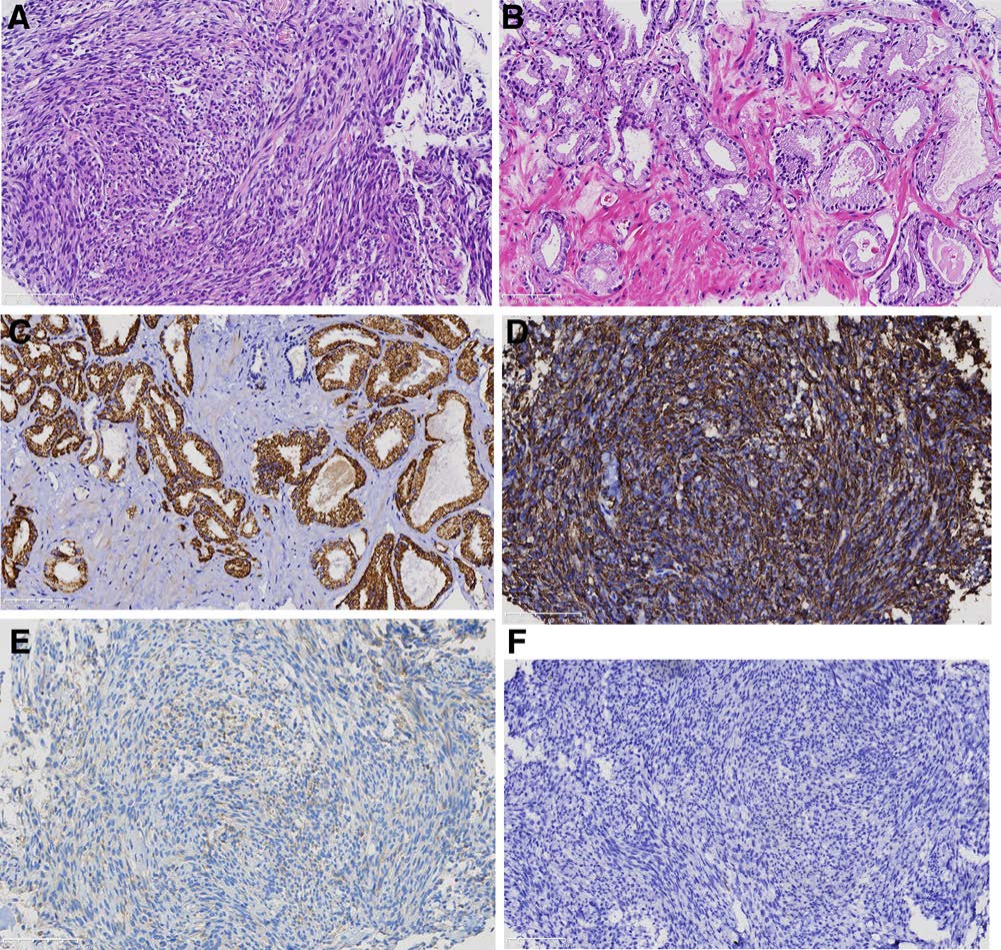
Pathological analysis of tumor tissue. (A) In some areas, the nuclei of tumor tissue are small and evenly spindle shaped, arranged in bundles (magnification: 200×). (B) In other areas, small glandular cavities are densely distributed, with enlarged nuclei and an increase in the ratio of nucleus to cytoplasm. Large and obvious nucleoli appear, without a basal cell layer (magnification: 200×). (C) Tumor cells are PSA positive in densely packed glandular cavities (magnification: 200×). (D–F) Spindle shaped tumor cells are EMA and BCL-2 positive, while CD117 negative (magnification: 200×). PSA = prostate specific antigen.

A 58-year-old male patient was found to have prostate space occupying lesions on physical examination, without obvious positive symptoms, special family tumor history and occupational hazard. Digital rectal examination showed that the prostate was Grade II hyperplasia, smooth in surface, hard in texture, without tenderness, Central sulcus disappeared, fingerstall were free of blood stain, and no abnormality was found in vulva and anus. The serum prostate specific antigen (PSA) level is normal. Prostate ultrasound examination showed an increase in prostate volume of 5.2 × 3.3 × 3.3 cm, mixed echo mass can be seen on the left side of the prostate, with a size of approximately 4.9 × 4.3 cm. The left seminal vesicle is compressed (Fig. 1). Prostate magnetic resonance imaging shows an increase in prostate volume, with T1 and T2 mass signals protruding to the left and rear in the left central zone of the prostate, and spotted high signal in diffusion weighted imaging (Fig. 2). Computer tomography did not show any occupying lesions in other areas, and no local lymph nodes or distant metastases were observed. Transrectal ultrasound guided prostate needle biopsy showed spindle cell tumors. The patient received radical prostatectomy, and histology showed that the nuclei in some areas of the tumor tissue were small and uniform fusiform, densely interlaced and arranged in bundles, with mild mitosis activity. In other parts of the region, the small gland cavity is densely distributed, the nucleus is enlarged, the proportion of nucleoplasm is increased, and large and obvious nucleoli appear, without stratum basale. Immunohistochemistry results showed positivity for TLE1, EMA, BCL-2, CKH, CK18, and Kiel-67 (in 46% of cells) in some regions of tumor cells; and negativity for Desmin, CD34, S-100, and CD117 in tumor cells. At the same time, tumor cells in the glandular ductal differentiation region showed P504S, PSA positive, and CKH, P63 negative (Fig. 3).These pathological results suggest the diagnosis of prostatic synovial sarcoma (biphasic type) combined with prostatic acinar adenocarcinoma, but still cannot provide sufficient diagnostic evidence.

Due to the rarity of the case, the pathological section was sent for second opinion and fluorescence in situ hybridization was performed on the tumor specimen. The results showed positive expression of the SYT-SSX fusion gene, confirming the diagnosis of primary prostatic synovial sarcoma (biphasic type) combined with prostatic acinar adenocarcinoma. The final histopathological examination showed that synovial sarcoma of the prostate involved about 30% of the glands, without lymphatic vessel infiltration or involvement outside the envelope. It was accompanied by acinar adenocarcinoma (Gleason 3 + 3), involving about 10% of the glands, and not involving the seminal vesicles.

There is currently no clear treatment guideline for this type of case. After discussion and consultation with a multidisciplinary diagnosis and treatment group, a comprehensive treatment plan for postoperative cyclic adjuvant chemotherapy (ifosfamide, Mesna, and doxorubicin) and radiotherapy has been unanimously determined. After continuous follow-up, at the time of this writing, the patient received 2 months of treatment according to this plan and no recurrence or metastasis was found.

## 3. Discussion

Synovial sarcoma is a rare subtype of fusion sarcoma that occurs in the prostate, mainly in young people with a median age of around 35 years old.^[3]^ Among all primary malignant tumors of the adult prostate, synovial sarcoma accounts for <0.1%.^[4]^ Synovial sarcoma can be divided into 4 types based on the proportion of epithelial and spindle cells: monophasic fibrous type: tumor cells are only composed of spindle cells; monophasic epithelial type: tumor cells are mainly composed of epithelial cells; biphasic type: tumor cells are composed of epithelial cells and spindle cell components mixed to varying degrees;^[5]^ poorly differentiated type: tumor cells are similar to small round cell tumors, with high-grade nuclear features and areas of necrosis.^[6]^ Monophasic spindle cells are the most common type.

Prostatic synovial sarcoma is a very rare soft tissue tumor, and cases of concurrent primary prostatic acinar adenocarcinoma are extremely rare. Due to nonspecific symptoms, its diagnosis is challenging and easily misdiagnosed. Differential diagnosis includes desmoid tumor, fibrosarcoma, carcinosarcoma, gastrointestinal stromal tumor, leiomyosarcoma, histiocytoma, mesothelioma, schwannoma, etc.^[7,8]^ Immunohistochemical and fluorescence in situ hybridization detection can assist in the diagnosis. The general pathology of synovial sarcoma is nonspecific and often presents as multi lobulated, accompanied by cysts, bleeding, necrosis, and calcification.^[9]^ Immunohistochemical analysis showed that EMA and BCL-2 were positive, while TLE1 was also a specific differentiation factor between synovial sarcoma and other types of sarcoma. Desmin and S-100 were negative.^[10]^ Immunohistochemical testing can exclude the possibility of other types of sarcoma in the prostate. In this case, the patient’s serum PSA level is within the normal range. Generally, the serum PSA level of prostate acinar adenocarcinoma increases. Due to the non-epithelial origin of synovial sarcoma, the patient’s serum PSA may not increase.

Prostate acinar adenocarcinoma is a malignant epithelial tumor composed of prostate secretory cells, with strong invasiveness. Most cases have multifocal growth, often located on the dorsal and dorsolateral sides of the prostate perizone. The general pathological manifestation is solid section, hard texture, large and deep stained nuclei, high nucleus-to-cytoplasm ratio, and cytoplasmic dichroism.^[11]^ Immunohistochemical shows that PSA and NXK3.1 are positive; P63, CKH, and P40 are negative, which is helpful for differential diagnosis.

Its rare incidence rate poses a challenge to the establishment of the best clinical treatment scheme. Up to now, almost all cases of prostatic synovial sarcoma or prostatic acinar adenocarcinoma have adopted the comprehensive treatment scheme of surgical resection combined with chemotherapy and/or radiotherapy. The size of the tumor and the involvement of surrounding soft tissues determine the degree of surgical intervention. Primary prostatic synovial sarcoma has a poor prognosis and is prone to metastasis. The most common cases are lung and lymph node metastasis.^[12]^ Postoperative clinical laboratory and imaging examinations are necessary, and regular follow-up is required.

## 4. Conclusion

Primary prostatic synovial sarcoma (biphasic type) combined with prostatic acinar adenocarcinoma is a very unique and rare case. To our knowledge, there are currently no reports of such cases, and effective treatment guidelines are not yet clear, which poses new challenges to clinical treatment. Early diagnosis and radical surgery combined with multidisciplinary treatment seem to be still a positive approach. With the further deepening of genetic research related to tumors, developing and improving targeted treatment strategies may become a feasible option for clinical treatment.

## Author contributions

**Conceptualization:** Yun Zhou, Longmei Wang.

**Data curation:** Qichong Shi.

**Formal analysis:** Qichong Shi, Yun Zhou, Longmei Wang.

**Investigation:** Qichong Shi, Yun Zhou, Longmei Wang.

**Project administration:** Yun Zhou.

**Resources:** Qichong Shi, Longmei Wang.

**Visualization:** Qichong Shi.

**Writing – original draft:** Qichong Shi.

**Writing – review & editing:** Qichong Shi, Longmei Wang.
